# The RiboMaP Spectral Annotation Method Applied to Various ADP-Ribosylome Studies Including INF-γ-Stimulated Human Cells and Mouse Tissues

**DOI:** 10.3389/fcvm.2022.851351

**Published:** 2022-03-28

**Authors:** Sasha A. Singh, Shiori Kuraoka, Diego Vinicius Santinelli Pestana, Waqas Nasir, Bernard Delanghe, Masanori Aikawa

**Affiliations:** ^1^Department of Medicine, Center for Interdisciplinary Cardiovascular Sciences, Brigham Women's Hospital and Harvard Medical School, Boston, MA, United States; ^2^Thermo Fisher Scientific (Bremen) GmbH, Bremen, Germany; ^3^Division of Cardiovascular Medicine, Center for Excellence in Vascular Biology, Brigham and Women's Hospital and Harvard Medical School, Boston, MA, United States; ^4^Channing Division of Network Medicine, Department of Medicine, Brigham and Women's Hospital and Harvard Medical School, Boston, MA, United States

**Keywords:** mass spectrometry, PARP14, post-translational modification, ribosylation, SEQUEST

## Abstract

ADP-ribosylation is a post-translational modification that is catalyzed by the ADP-ribosyltransferase enzyme family. Major emphasis to date has been ADP-ribosylation's role in cancer; however, there is growing interest in its role in inflammation and cardiovascular disease. Despite a recent boom in ADP-ribosylation mass spectrometry-based proteomics, there are limited computational resources to evaluate the quality of reported ADP-ribosylated (ADPr) proteins. We recently developed a novel mass spectral annotation strategy (RiboMaP) that facilitates identification and reporting of ADPr peptides and proteins. This strategy can monitor the fragmentation properties of ADPr peptide-unique fragment ions, termed m-ions and p-ions, that in turn provide spectral quality scores for candidate ADP-ribosyl peptides. In this study, we leveraged the availability of publicly available ADP-ribosylome data, acquired on various mass spectrometers, to evaluate the broader applicability of RiboMaP. We observed that fragmentation spectra of ADPr peptides vary considerably across datasets; nonetheless, RiboMaP improves ADPr peptide spectral annotation across all studies. We then reanalyzed our own previously published *in vitro* ADP-ribosylome data to determine common responses to the pro-inflammatory cytokine, IFN-γ. We conclude that despite these recent advances in the field of ADPr proteomics, studies in the context of inflammation and cardiovascular disease still require further bench-to-informatics workflow development in order to capture ADPr signaling events related to inflammatory pathways.

## Significance

Mass spectrometry-enabled ADP-ribosylation workflows are developing rapidly, providing researchers a variety of enrichment strategies and mass spectrometric acquisition options to identify ADP-ribosylated proteins (ribosylome). In order to accommodate this growing field computatonally, we developed a novel mass spectral annotation strategy (RiboMaP) that facilitates identification and reporting of ADP-ribosyl peptides and proteins. We demonstrated that RiboMaP can analyze any type of higher collision energy dissociation (HCD)-dependent ribosylome data, demonstrating that this software has broad applicability.

## Introduction

ADP-ribosylation (ADPr), or ribosylation, of proteins is intensively being studied in the context of DNA damage and cancer therapies (1, 2). Proteomics studies have capitalized on hydrogen peroxide's strong induction of DNA damage and downstream ribosylation events to achieve the ribosylome yields, 10-fold above physiological levels (3), necessary for enrichment and mass spectrometric method optimizations (4–6). More recent studies have investigated the role of ribosylation in cellular processes contributing to cardiovascular disease, such as vascular inflammation (7, 8), host-pathogen interactions (8, 9) or calcification (10). We, for instance, have studied the changes to the ribosylome in response to the pro-inflammatory cytokine IFN-γ in both a human cell culture (11) and a mouse model for acute inflammation (12).

In the last decade, several workflows to enrich ribosylated proteins/peptides for mass spectrometric analysis have been developed. We and others have implemented an enrichment strategy that retains a mono-ADPr (MAR) moiety on peptides (13). Specifically, a laboratory-engineered poly-ADPr (PAR) and MAR-binding macrodomain from *Archaeoglobus fulgidus* (eAf1521) (14) can be used to enrich ADPr peptides from a proteolyzed cell or tissue sample. Since this ADPr peptide pool contains both PAR and MAR, and only the latter is conducive to mass spectrometric analysis, a step that converts PAR to MAR peptides using poly-ADP-ribose glycohydrolase (PARG) is required (13). Additionally, anti-ADPr antibody-based enrichments can also be employed since they also produce MARylated peptides when PARG is applied (15). MARylated peptides produce diagnostic MS2 fragment ions (more below) that can be used to perform a type of ‘smart sequencing’ for ADPr peptides by the mass spectrometer. Specifically, this sequencing strategy entails a higher collision energy (HCD) scan to screen for diagnostics ADPr fragments, that if detected, triggers additional dissociation methods on the same ADPr peptide to maximize sequencing (11, 12). Smart sequencing is beneficial since despite being enriched, ADPr peptides comprise <50% of the total peptide pool (12).

When using higher collision energy dissociation (HCD) to fragment ADPr peptides for sequence identification, diagnostic fragment (MS2) ions are produced: the low mass fragments from the ADP-ribose moiety (m-ions) and the complementary peptide plus remaining ADP-ribose fragment (p-ions) (16). The MS2 spectra therefore comprise the typical fragments (termed b-ions and y-ions) for peptide identification, but also the ADPr unique m-ions and p-ions that are not readily recognized by standard spectral annotation software. We recently developed a bioinformatic strategy that annotates and scores ADPr peptides' m-ions and p-ions (12). We demonstrated that this method could increase the number of reportable mouse-derived ADPr peptides.

In this study, we applied our ADP-ribosyl peptide m-ion and p-ion annotation method, termed RiboMaP, to ribosylation spectra collected from other laboratories. We demonstrate that RiboMaP's m-ion and p-ion scores provide insight into the dissociative properties of ADPr peptides sourced from various experimental conditions; and that despite these differences, RiboMaP can increase the number of reportable ADPr peptides. We then re-analyzed our previously published data to explore whether IFN-γ induced common changes to the ADP-ribosylome in a human cell culture system vs. mouse liver and spleen.

## Methods

### ADPr Peptide Mass Spectral Datasets

To demonstrate the broad applicability of RiboMaP, we downloaded publicly available ADPr proteomic datasets (PRIDE database: https://www.ebi.ac.uk/pride/). These data include ADPr peptides from mouse liver analyzed by the Q Exactive-HF [PXD004245] (17); hydrogen peroxide-treated human cervical cancer cell line HeLa analyzed by the Orbitrap Fusion [PXD004767] (6); IFN-γ-exposed mouse liver analyzed on the Orbitrap Fusion Lumos and the Q Exactive [PXD024580] (12); and IFN-γ-treated human macrophage-like cell line THP-1 analyzed by the Orbitrap Fusion Lumos [PXD0115690] (11).

### Annotation of ADPr Peptide Spectra

HCD mass spectra were analyzed using a customized ADPr annotation and scoring module (termed RiboMaP) developed internally as an enhancement for Proteome Discoverer (PD version 2.4, Thermo Fisher Scientific) (12). The mass spectral acquisition method for each downloaded dataset was confirmed from a representative.RAW files. The spectra were queried against the Uniprot mouse (*n* = 63,703 entries) or human (*n* = 96,816 entries) fasta database (both downloaded September 09, 2020) using the SEQUEST search engine algorithm. Trypsin was set as the digestion enzyme, allowing up to 4 missed cleavages and a minimum peptide length of 6 amino acids. ADPr (+541.061 Da) of Asp, Glu, Lys, Arg, Ser, Thr, Tyr and His; oxidation (+15.995 Da) of methionine; and acetylation (+42.011 Da) of the N-terminus, were set as variable modifications. Carbamidomethylation (+57.021 Da) of cysteine was set as a static modification. Spectral search tolerances were 10 ppm for the precursor mass and 20 mmu for the MS2. The peptide false discovery rate (FDR) was calculated using Percolator (target/decoy method, separate databases) and peptide spectrum matches (PSMs) were filtered based on a 1.0 or 5.0% FDR, as indicated. Typically, an m-series score >30 indicates a true ADPr peptide spectrum, and the p-series score validates candidate spectra identified by SEQUEST (12, 18) (Supplemental Information). All ADPr peptide spectral matches and their corresponding spectral features and scores (including the m-series and p-series scores) (12) are included in Supplementary Tables S1–S5.

## Results

### ADPr Peptide Dissociation Properties and Datasets Analyzed in This Study

**Figure 1:**
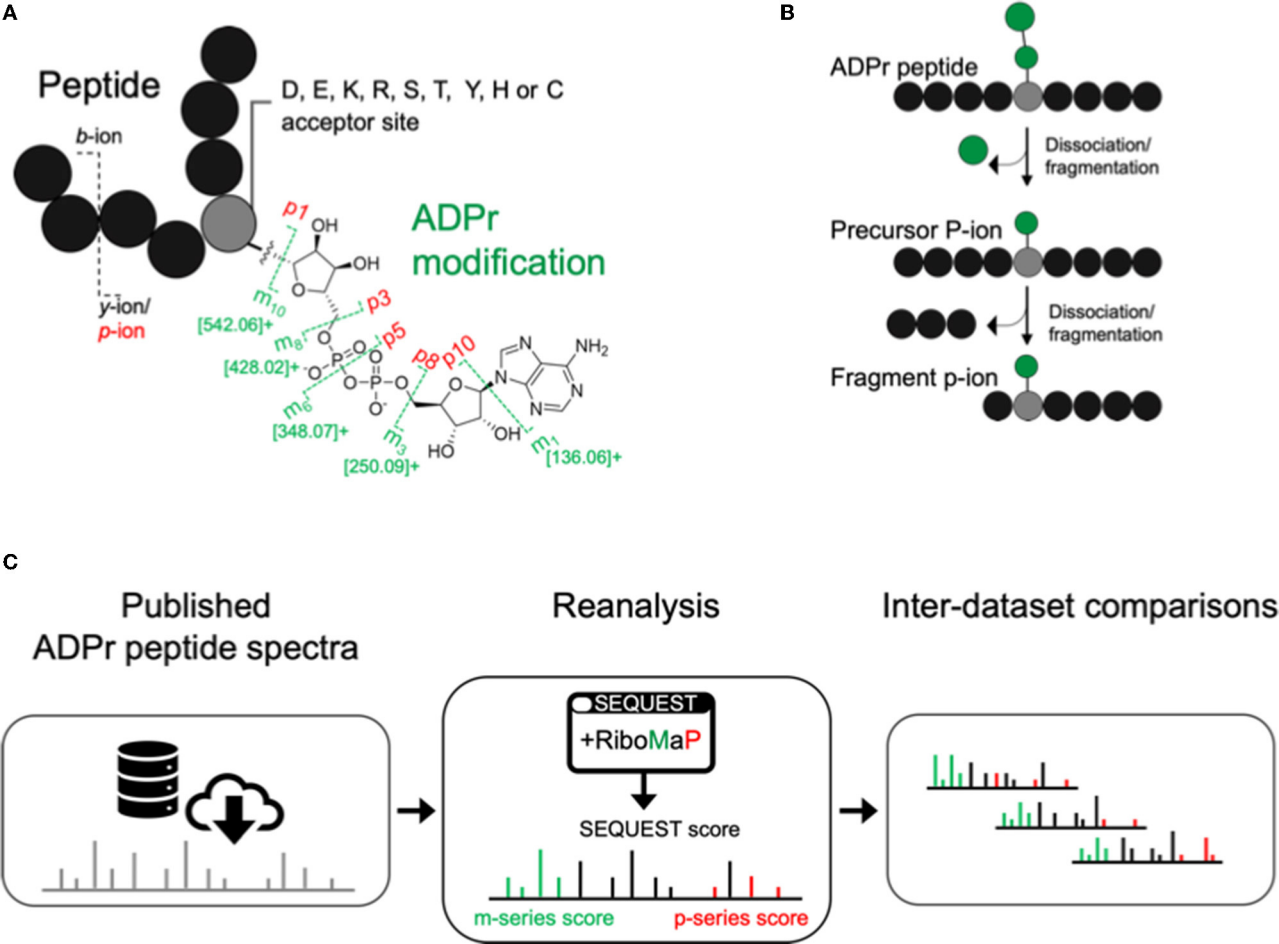
Inter-study analysis using a single workflow to annotate ADP-ribosyl data. **(A)** The ADP-ribosyl (ADPr) post-translational modification with its major higher collision energy (HCD) dissociation products (annotated as the m- and p-ions). **(B)** Schematic depicting sequential dissociation that converts precursor P-ions to fragment p-ions. **(C)** Workflow for ADP-ribosylome data re-analysis using RiboMaP.

Higher collision energy dissociation (HCD) fragments the ADPr peptide amide backbone into b-ion and y-ions, and the ADPr modification into m-ions and p-ions (Figure 1A) (6). The p-ions themselves are further classified as precursor P-ions (intact peptide with residual modification) and fragment p-ions (further dissociation of the precursor into fragments with the residual modification) (Figure 1B) (12). We downloaded ADP-ribosylome datasets that represent various experimental conditions, that have now been analyzed by a common spectral annotation workflow. This workflow comprises our recently developed RiboMaP that supplements the search engine's (SEQUEST) PSMs with m-ion and p-ion annotations (Figure 1C).

### The m-ion and p-ion Series Scores Indicate That ADPr Peptides Fragmentation Properties Vary Across Mass Spectrometers

**Figure 2:**
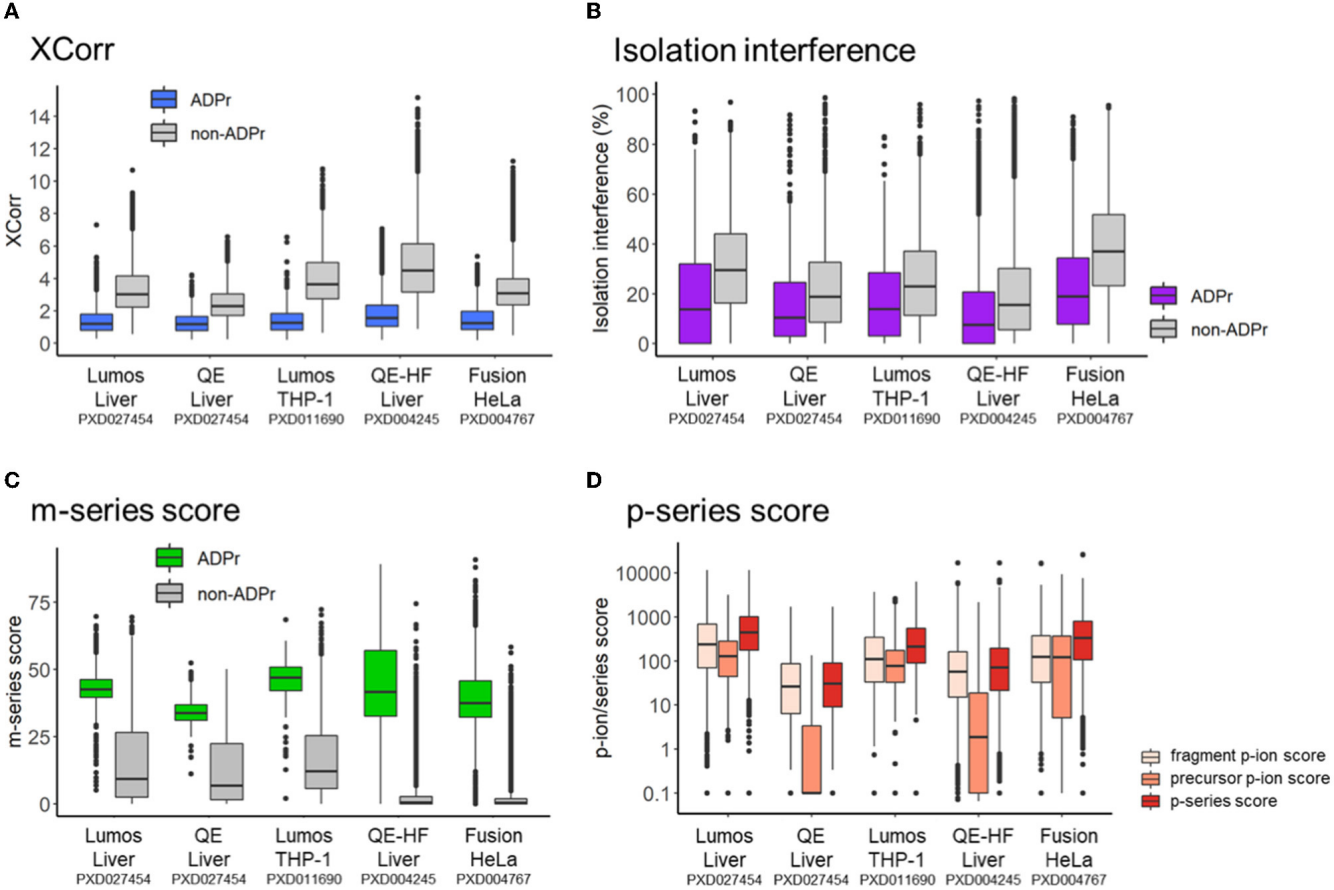
Intra- and inter-dataset comparisons of ADP-ribosyl peptide spectral matches. **(A)** XCorr, the cross-correlation score between a spectrum and database match. **(B)** Isolation interference, the relative amount of ion current within the isolation window that is not attributed to the isolated precursor. **(C)** m-series score, the scaled total absolute m-ion intensity normalized by base peak intensity. **(D)** p-series score, total absolute p-ion intensity normalized by base peak intensity.

We previously demonstrated that the same pool of ADPr peptides analyzed by different mass spectrometers (i.e., the Lumos vs. the Q Exactive) exhibits common and distinct fragmentation properties (12). In this study, we first compared the search engine score (XCorr) for ADPr vs. non-ADPr PSMs across the five datasets (Figure 2A). The XCorr values for ADPr PSMs are consistently lower than the non-ADPr PSMs sequenced in the same mass spectrometric analysis; for example an XCorr median of 1.19 (ADPr) vs. 3.02 (non-ADPr) for the Lumos liver dataset (Figure 2A). Secondly, ADPr PSMs require lower interfering signal from co-isolated peptides (isolation interference) to be confidently identified by the search engine. Across the five datasets, the median isolation interference is consistently below 20% for ADPr PSMs, and 20% or above for non-ADPr PSMs (Figure 2B).

Differences across the datasets are apparent when comparing m-series and p-series scores. Although the median m-series score is at least 30 for all instrument datasets, the 50^th^ percentiles vary: the Q Exactive on the lower end (m-series score median = 33.8 for a liver dataset) and the Lumos on the upper end (m-series score median = 46.9 and 42.5 for THP-1 and liver datasets, respectively) (Figure 2C). It is worthy to note that non-ADPr spectra also contain m-ions, but their scores are low since the presence of m-ions are due to the stochasticity of ADPr peptide co-isolation (12). Relative to the m-series, the p-series scores are more complex, reflecting sequential fragmentation of a precursor P-ion into fragment p-ions (Figure 1B) (12). RiboMaP provides scores for all three, fragment p-ions, precursor P-ions, and both combined (p-series score) (Figure 2D). In this case, the Q Exactive and the Q Exactive-HF datasets score lower for the p-series ions, with the precursor P-ion the lowest (Figure 2D). We previously demonstrated that as collision energy increases on the Q Exactive, the p-series scores, especially the P-ion score, decrease drastically as compared to the Lumos (12). This current extended inter-dataset analysis thus underscores that ADPr peptide fragmentation can vary considerably.

### The ADPr Modification Dissociates Independently of the Peptide Backbone

**Figure 3:**
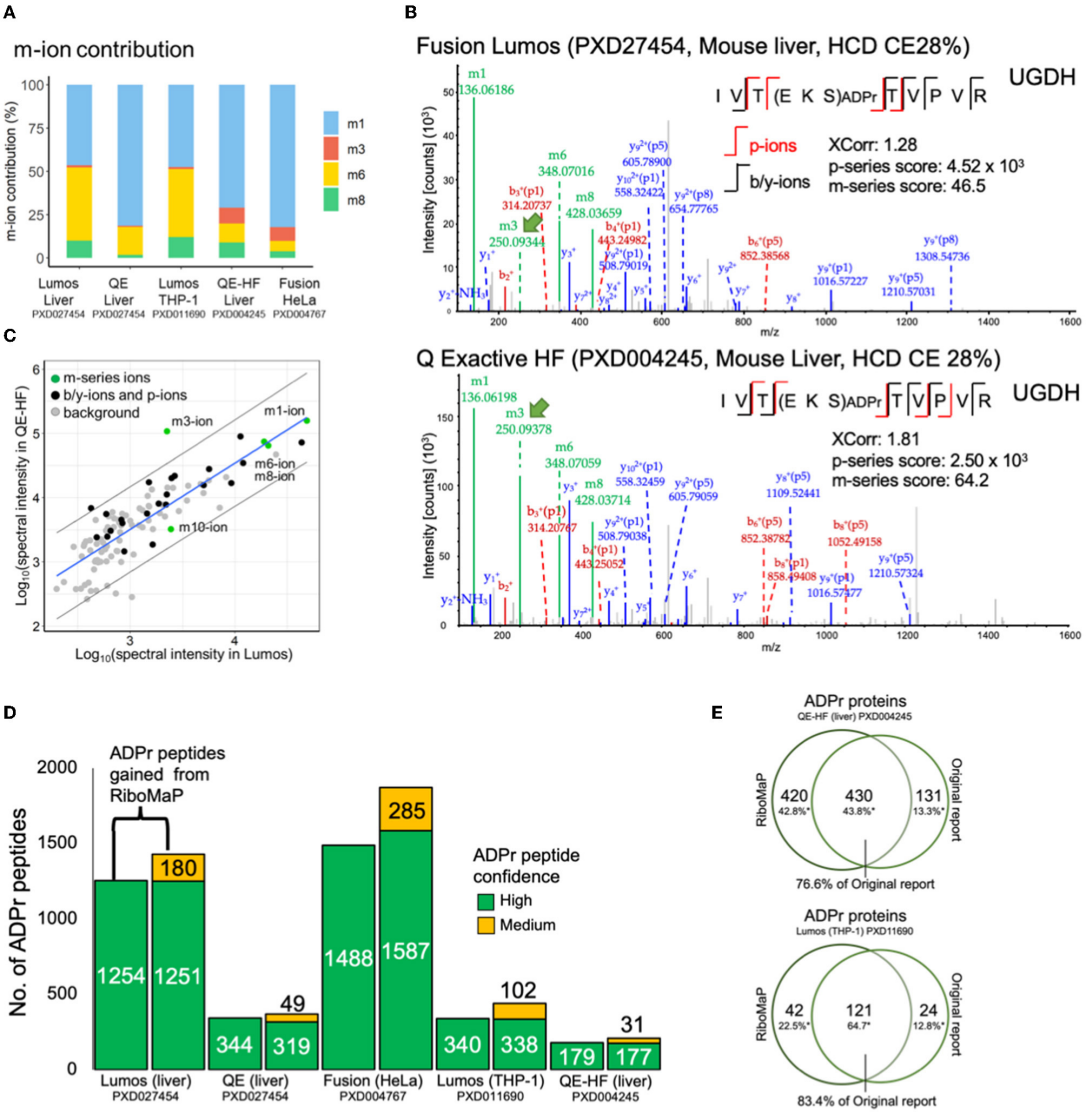
Applying RiboMaP to increase the number of ADP-ribosyl peptide spectral matches (PSMs). **(A)** A breakdown of the contribution of individual m-ions to the m-series score. **(B)** An ADPr PSM identified in two independent studies contains similar fragment ions. UGHD, UDP-glucose 6-dehydrogenase. **(C)** A spectrum-to-spectrum peak intensity plot from panel B shows that the m3-ion intensity is markedly higher in the QE-HF compared to the Lumos. **(D)** A comparison between default and RiboMaP-enabled reporting of ADPr peptides. **(E)** Our current workflow accounts for the majority of ADPr proteins reported previously for the QE-HF liver study (76.6%) and the Lumos THP-1 study (83.4%).

We examined further the m-series scores (Figure 2C) and confirmed that the prevalence of individual m-ions varies across the datasets (Figure 3A). RiboMaP specifically scores the m1, m3, m6 and m8-ions (Figure 1A), since they are the most intense, but others exist such the m10-ion corresponding to the entire ADPr modification (12, 19). The m1-ion (adenine ring) is the dominant fragment regardless of instrument, followed by the m6-ion on the Lumos and Q Exactive, and then by a more equal occurrence of the m3, m6, m8-ions on the Fusion and the QE-HF (Figure 3A). Although m-ions can be formed by the dissociation of the ADPr peptide itself, the m1 and m3-ions likely also form from further dissociation of one or more of the larger m-ions (12).

The increased prevalence of the m3-ion in Fusion (HeLa) and QE-HF (liver) data may therefore be due to dissociation of an already dissociated ADPr moiety, rather than increased dissociation of the m3/p8 bond of the ADPr peptide itself (Figure 1A). To investigate this further, we extracted an ADPr PSM acquired from each dataset to compare fragment ions (Figure 3B). The spectra are largely similar, but a notable difference is the high m3-ion signal in the QE-HF scan (Figure 3B, green arrow). For clarity, we extracted the commonly identified fragment ions between the Lumos and QE-HF scans and plotted their relative rank intensities (Figure 3C). Most fragment ions' intensities are within the 95% confidence interval (with the m1, m6 and m8-ions perfectly correlated), except for the m3-ion with higher intensity in the QE-HF scan (Figure 3C). Despite its increased intensity, we did not detect the complementary P8/p8-ion in the QE-HF scan, suggesting that this increase in m3-ion intensity is due to dissociation of the ADPr modification. Interestingly, the ADPr/m10-ion's intensity in the QE-HF scan is lower than that of the Lumos indicating relative signal loss due to its increased dissociation (Figure 3C). What these data emphasize is that the m-ion intensities do not necessarily indicate the prevalence of their complementary p-ion ions, preventing the use of the canonical m/p-ion complementarity to predict the p-series fragments based on the m-series fragments.

### RiboMaP Increases the Number of Reportable ADPr Spectra in Previously Published Studies

The p-ions provide additional support for the candidacy of ADPr PSMs. The p-series score is important given that ADPr PSMs' XCorr values are lower than those of typical peptides (Figure 2A). For instance, we previously established that a p-series score of at least 10 for peptides with charge states of 2+ and 3+, and at least 100 for charges states 4+ and greater could be used to support ADPr PSMs with lower confidence (Supplementary Figure S1A). If using criteria typical of standard proteomics experiments, only high confidence PSMs (1% FDR) would be reported. The availability of the p-series ions therefore permits the inclusion of several medium confidence PSMs (5% FDR cut-off), although at the cost some high confidence ADPr PSMs (Supplementary Figure S1B). With the inclusion of RiboMaP, we increased the number of ADPr reportable peptides by including the medium confidence ADPr peptides whose PSMs were supported by the p-series score, but we also lost some high confidence peptides (Figure 3D). For example, in the Lumos liver dataset, 1,254 high confident peptides were generated from the default workflow, but once RiboMaP was implemented, that number dropped slightly to 1,251; however, we gained 180 medium confident peptides as a trade-off resulting in a 14% increase in ADPr peptides (Figure 3D). The net gain in ADPr peptides ranged from 6% for the QE liver data to 29% for the Lumos THP-1 data (Figure 3D). Since the mouse liver QE-HF protein identifications were published, we compared them to those we identified; and despite the different search engine and spectral analysis workflow used in the original report (17) our workflow accounted for 76.6% of the previously reported ADPr proteins (Figure 3E). Similarly, despite the difference in the spectral analysis workflow, 83.4% of the proteins were accounted for in our previously reported THP-1 ADPr proteome study (Figure 3E) (11).

### Integration of ADP-Ribosylomes From Three IFN-γ Stimulation Experiments

**Figure 4:**
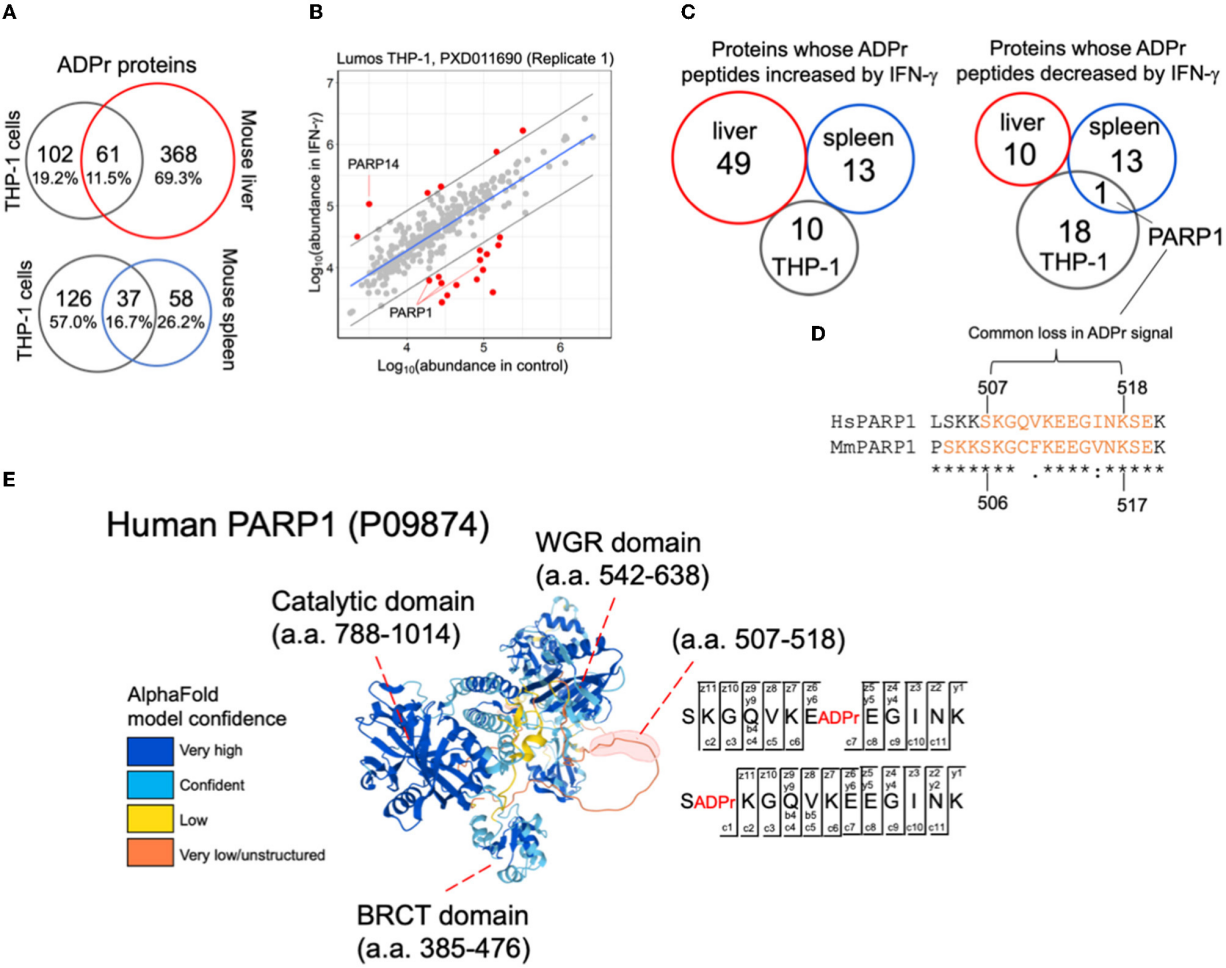
A comparison between IFN-γ treatment studies. **(A)** Common and unique ADPr proteins between human THP-1 cells, and mouse liver or spleen. **(B)** A linear model plot using R. The gray lines indicate the 95% confidence intervals. **(C)** Comparisons across studies demonstrate no overlap for IFN-γ effects on the ADP-ribosylomes except for a common sequence in PARP1. **(D)** PARP1 amino acid sequence determined to be commonly ADP-ribosylated in human THP-1 cells and mouse spleen datasets. The orange amino acids correspond to sequence coverage in THP-1 and mouse PARP1 data. The peptide whose ADPr signal decreased in both THP-1 and mouse spleen data is indicated. **(E)** The IFN-γ modulated PARP1 ADPr peptide resides within a predicted disordered domain. Both glutamate and serine amino acids were identified (EthcD annotation provided) to be ADP-ribosylated in THP-1 cells (and mouse spleen) (Supplementary Figure S3).

With an improved understanding of ADPr peptide spectral features than what we knew at the time of reporting the THP-1 IFN-γ stimulation study (11), we re-analyzed these data to compare them with the mouse IFN-γ injection study, that includes ADPr peptides prepared from whole liver and whole spleen (Supplementary Table S6). There is a greater overlap between THP-1 and spleen (16.7%) as compared to THP-1 and liver (6.4%), despite the higher number of ADPr proteins identified in liver (Figure 4A). This greater overlap with spleen is not surprising since the organ is rich in monocytes and macrophages (20, 21).

We then explored the INF-γ-induced changes in common across THP-1 cells, mouse liver and mouse spleen. For THP-1 cells, we acquired two independent biological replicates months apart (with 15 culture plates per IFN-γ and control condition). Each replicate exhibited distinct data distributions (11) thus we re-analyzed them separately (Figure 4B; Supplementary Figure S2; Supplementary Table S6). As previously noted, ADPr peptides from IFN-γ-inducible PARP14 (Figure 4B) and PARP9 (Supplementary Figure S2) increased with IFN-γ; but PARP1 ADPr peptides decreased (Figure 4B; Supplementary Figure S2). When we compared all differentially abundant ADPr peptides from human THP-1 cells with those reported from mouse liver and spleen (12), none commonly increased with IFN-γ between mouse and THP-1; and only PARP1 appeared as commonly decreased in mouse spleen and THP-1 (Figure 4C; Supplementary Tables S7, S8). Interestingly, the latter includes the sequence 507-SKGQVKEEGINK-518 from THP-1 that overlaps with 509-GAVKEEGINKSE-521 from mouse spleen (Figure 4C). This sequence is located between the BRCA1 C Terminus (BRCT) and Trp-Gly-Arg (WGR) domains of PARP1, specifically within a predicted disordered domain (Figure 4D) that was previously reported to be ADP-ribosylated, primarily through PARP1's auto-ribosylation activity (22).

Specifically, glutamate-513 (SKGQVKE(ADPr)EGINK) from human THP-1 cells and serine-520 (GAVKEEGINKS(ADPr)E) from mouse spleen decreased in IFN-γ condition (Figure 4E; Supplementary Figure S3). Although the decrease in ADPr signal for each peptide suggests a decrease in the occupancy of ADP-ribosylation, it is important to note that PARP1 itself decreases, although subtly, in response to IFN-γ (11, 12). It is also noteworthy that a common serine was identified to be ADP-ribosylated in human THP-1 cells and mouse spleen (serine-507 (S(ADPr)KGQVKEEGINK; Figure 4D; Supplementary Figure S3); and that this serine was either unchanged (mouse spleen) or increased slightly (THP-1) in response to IFN-γ, that when considering the decrease in total PARP1, may indicate an increase in ADP-ribosylation status at this serine.

## Discussion

Ribosylation enrichment protocols and mass spectrometry acquisition methods have been intensely investigated, however informatics methods that address the complexity ADPr peptide fragmentation data did not exist until RiboMaP (12). Our interests in ADP-ribosylation in macrophage biology and proinflammatory signaling (7, 8, 11) led us to develop RiboMaP; primarily since our first ever study into an ADP-ribosylome (THP-1 cells stimulated with IFN-γ) revealed lower spectral scores for ADPr peptides, exemplifying the need for ADPr peptide standards to validate ADPr peptides of interest (11). Moreover, we had to manually annotated the P/p-ions that were not recognized by the search engine (11).

In this current study, we reinvigorated the THP-1 INF-γ study by reanalyzing the mass spectral data but this time with the aid of RiboMaP; increasing the number of ADPr peptides from 340 to 450 (Figure 3D). However, despite commonly identified ADPr proteins between human THP-1 cells and mouse tissues, there was virtually no overlap in terms of IFN-γ-induced changes other than a loss of ADPr signal for PARP1 in spleen and THP-1 cells. It therefore would be more appropriate to compare THP-1 cells with a more comparable cell source, such as human primary or mouse primary monocytes/macrophages. However, it remains challenging to perform ADP-ribosylation proteomics on such lower yielding circulatory compartments compared to whole tissues (23).

Additionally, most studies to date have examined the potent changes to ribosylation signatures induced by hydrogen peroxide (17, 22); whereas IFN-γ is relatively subtle, inducing changes that are more challenging to quantify (11). PARP1 is very abundant thus increasing the chance of detecting and quantifying ADP-ribosylation signatures. Although a net decrease in PARP1 ADP-ribosylation was observed in THP-1 cells and spleen INF-γ conditions, we cannot rule out that this decrease is due to the decrease in PARP1 itself (11, 12).

On a broader scale, we applied RiboMaP to publicly available data, to evaluate the dissociation (fragmentation) properties ADPr peptides across various datasets. Understanding variables that impact dissociation will help improve further RiboMaP. Specifically, we are actively investigating statistical-based validations for the p-series score cut-off. That is, the p-series thresholds that we currently employ (Supplementary Figure S1) are based on XCorr thresholds more suitable for typical (non-ADPr) peptides and p-series thresholds based on manual validation (12). To incorporate m-ion and p-ion features into a machine learning approach, such as the one provided by Percolator (24), we need to examine the range of ADPr peptide dissociation properties to develop a validation strategy suitable for all experimental types. We therefore need to determine the most relevant m-series and p-series ions. This feature selectivity is particularly important for the p-series since the various P/p-ions combinations greatly outnumber the b/y-ions, risking an increase in the false-discovery rate for the reverse-decoy database search in Percolator.

Our ongoing studies will therefore establish which of the m-ions and P/p-ions are representative of all experiment types, and which most accurately reflect dissociation of the ADPr peptide itself (e.g., m6-p5), and not sequential dissociation of the ADPr moiety that generates the predominant m1-ion. In parallel, we can continue to optimize ADPr peptide/protein enrichment protocols to capture lower abundant ADPr proteins that may provide more insight into the precise roles ribosylation plays in immunity and inflammation.

## Data Availability Statement

The datasets presented in this study can be found in online repositories. The names of the repository and accession number(s) can be found in the article.

## Ethics Statement

All animal procedures used in this study were approved by and performed in compliance with Beth Israel Deaconess Medical Center's Institutional Animal Care and Use Committee (protocol #: 021-2017).

## Author Contributions

SS, SK, and DP performed data analysis. WN and BD developed the software and advised on data analysis. SS and SK conceptualized the study and wrote the main manuscript. MA supervised the study and provided resources and funding. All authors contributed to the editing of the manuscript.

## Funding

This study was in part supported by research grants from Kowa Company, Ltd., Nagoya, Japan, and the National Heart Lung and Blood Institute (R01HL126901 and R01HL149302 to MA). Other than funding, the study did not involve Kowa.

## Conflict of Interest

SK is an employee of Kowa Company., Ltd., and was a visiting scientist at Brigham and Women's Hospital and Harvard Medical School when the study was conducted. BD and WN are employees of Thermo Fisher Scientific. This study was in part supported by research grants from Kowa Company, Ltd. Nagoya, Japan, and the National Heart Lung and Blood Institute (R01HL126901 and R01HL149302 to MA). The funder was not involved in the study design, collection, analysis, interpretation of data, the writing of this article or the decision to submit it for publication. The remaining authors declare that the research was conducted in the absence of any commercial or financial relationships that could be construed as a potential conflict of interest.

## Publisher's Note

All claims expressed in this article are solely those of the authors and do not necessarily represent those of their affiliated organizations, or those of the publisher, the editors and the reviewers. Any product that may be evaluated in this article, or claim that may be made by its manufacturer, is not guaranteed or endorsed by the publisher.
