# Supplementary material for: The RiboMaP Spectral Annotation Method Applied to Various ADP-Ribosylome Studies Including INF-γ-Stimulated Human Cells and Mouse Tissues
Source: Front Cardiovasc Med. 2022 Mar 28;9:851351. doi: 10.3389/fcvm.2022.851351 (PMC8996112; doi:10.3389/fcvm.2022.851351)
Supplement: Supplementary file 7 [file Data_Sheet_1.pdf]

A

IFN-γ enriched
- PARP14
- LAMTOR3
- DSTN
- HSP90AB1
- RPS27A
- RPS27A

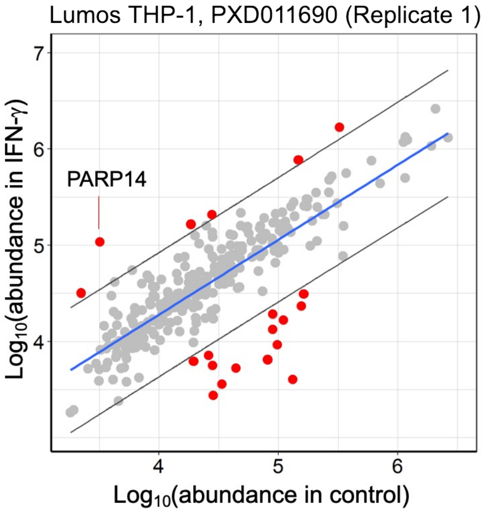

Control enriched
- HMGA1
- PARP1
- TRIM11
- NPM1
- ACTB
- PARP1
- PARP1
- PARP1
- TMSB4X
- MYH9
- TMSB4X
- TMSB4X
- HNRNPU
- TPM3

B

IFN-γ enriched
- PARP14
- RPS27A
- CAP1
- RPS27A
- PARP9
- ACTB
- RNF114
- RPS27A
- RPS27A
- PARP1

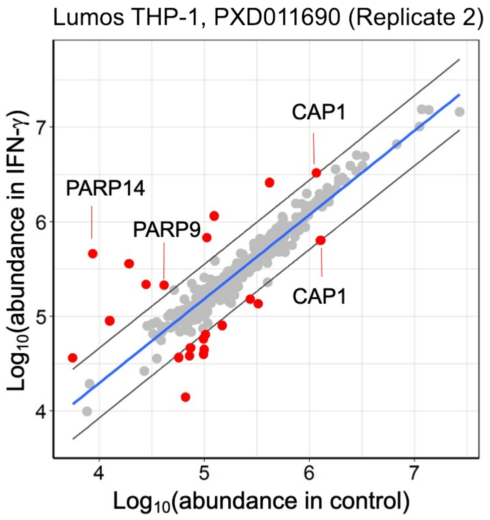

Control enriched
- MORC1
- CREBZF
- MYH9
- FABP5
- ACTN4
- H2BC21
- CDH12
- TPM3
- ATAD3A
- RFC1
- ATG16L2
- CAP1

Supplemental Figure S2. Correlation plot of relative ADPr peptide abundance between replicates 1 **(A)** and 2 **(B)** from (Higashi et al., 2019). The ADPr peptide points are the combined quantification from 1 full scan + 5 gas phase segmented scans, for each IFN-γ and control condition. Red points are outside proteins whose ADPr peptides are outside the 95% confidence interval of the linear model (plotted in R). These outlier proteins are listed.

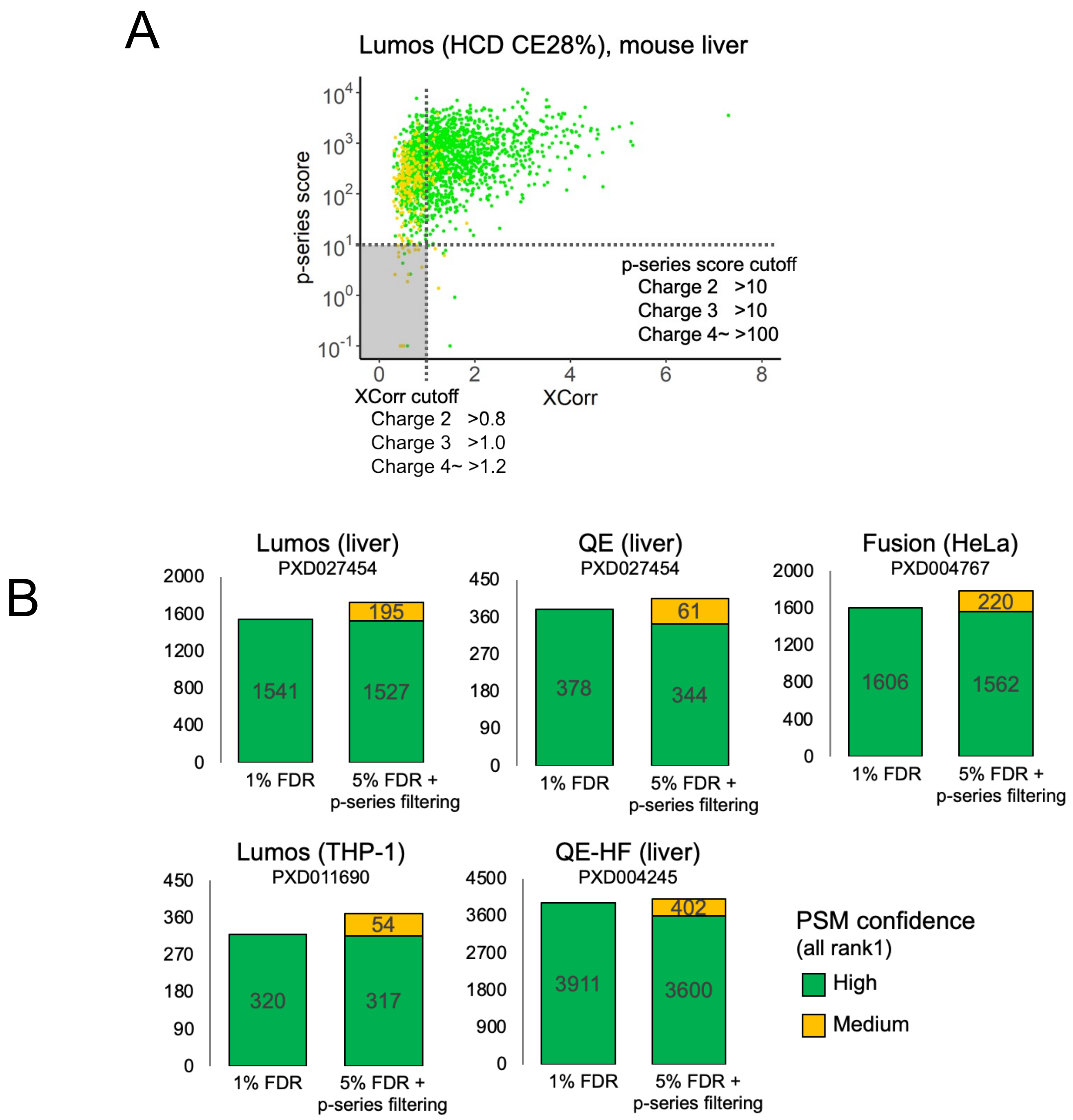

Supplemental Figure S1. (A) Scatterplot of the relationship between XCorr and the p-series score. The XCorr cut-offs are recommended from Percolator/Proteome Discoverer. The p-series score cut-offs are recommended from manual inspection of the data. **(B)** Peptide spectrum matches (PSMs) resulting from standard proteomics experimental filters (high confidence, rank1 PSMs only) vs those resulting when the p-series score is considered (including both high and medium rank1 PSMs).

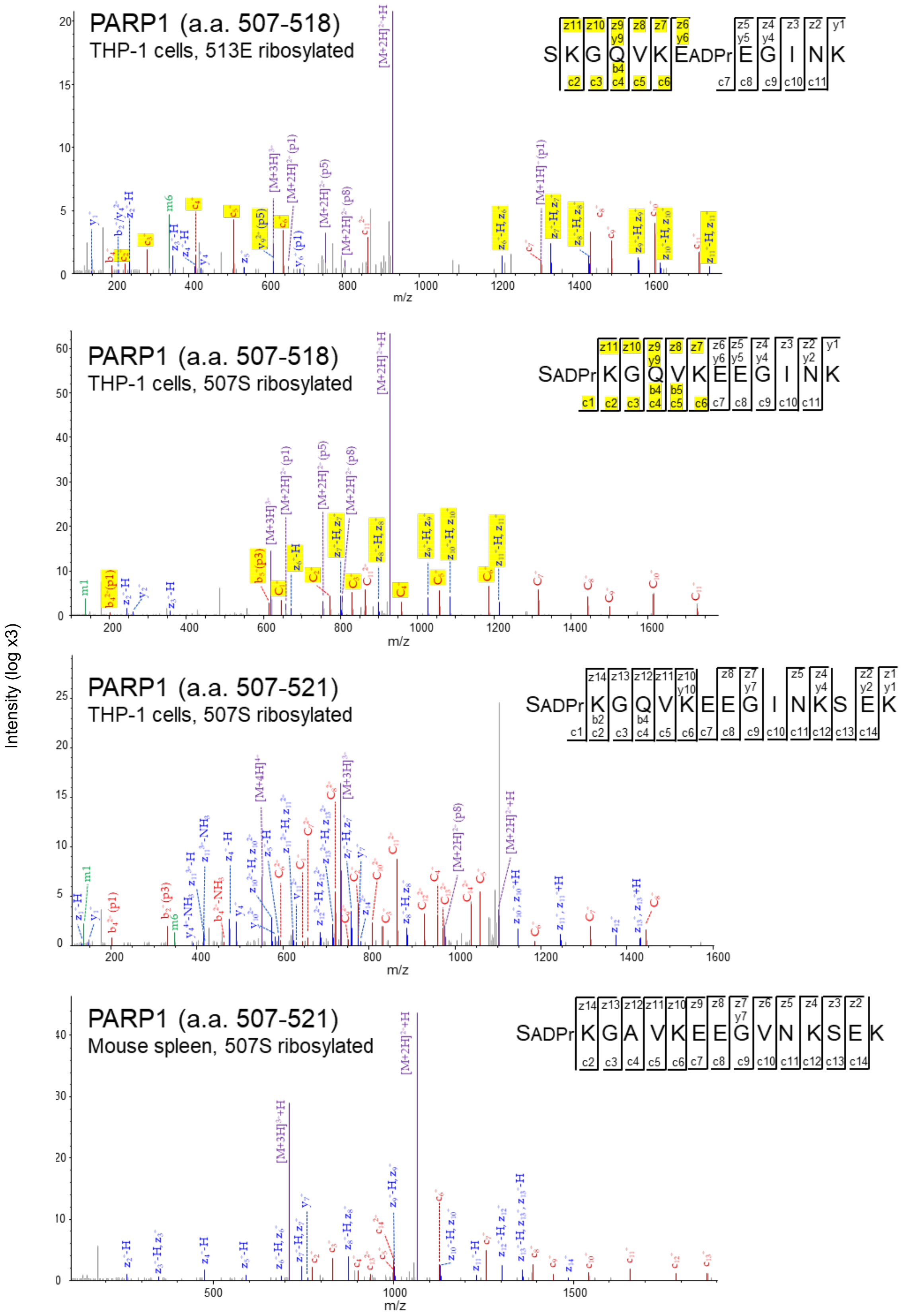

Supplemental Figure S3. EThcD MSMS spectra of PARP1 peptides from THP-1 cells (Higashi et al., 2019) and mouse spleen (Kuraoka et al., 2021). Highlighted c-ion and z-ions differentiate serine and glutamate acceptor sites.
